# A Practical Diet Table for Diabetics

**Published:** 1879-04

**Authors:** 


### A PRACTICAL DIET TABLE FOR DIABETICS.

A distinguished physician of this city has handed us the following bill of fare, which is the result of the experience of a sufferer from diabetes, to whom these foods have proved not only unobjectionable, but conducive to a cure. In view of the fact that the highest medical authorities have decided that medicines are of little or no avail in this disease; that the chief reliance is upon appropriate food; that improper food surely encourages the disease, while suitable foods unfailingly retard its progress, and that very few who suffer from this trouble are accurately informed as to what foods are admissible and what are objectionable or dangerous, he offers this list, as containing nothing which has proved injurious in his own case.

The suggestions in regard to bathing, exercise, and most of the foods, are the results of his consultations with eminent medical men in America and Europe, among whom may be mentioned Professor Bouchardat, of Paris; and their value as curative agents are borne out in his own experience.

As the elimination of sweets and starches has proved beneficial in nervous prostration and brain exhaustion, it is believed that the following bill of fare may be wisely adopted by all nervous sufferers:

*Oysters and Clams*—Raw, or cooked without flour mixtures.

*Soups.*—All those without flour, rice, vermicelli or other starchy substances, or the prohibited vegetables.

*Fish*—Of all kinds, including lobsters and crabs.

*Meats*—Of all kinds, more particularly beef and mutton (liver not used); also tripe, ham, bacon and sausages.

*Poultry and Game*—Of all kinds. Avoid sweet jellies and sauce with the game.

*Salads*—In all varieties except potato. Use freely of spinach, lettuce, watercress, Brussels sprouts, chiccory, dandelions and cold slau; also olives. Cabbage in all forms especially valuable.

*Vegetables*—Of all kinds except potatoes, beets, carrots, turnips, parsnips, peas, beans, rice, or those containing sugar or starch. Young onions have been found particularly valuable. Sour apples, cut in quarters, dipped in beaten eggs, rolled in cooked gluten, and fried in very hot fat, make a good substitute for potatoes.

*Fruits.*—All kinds of tart fruits. Peaches and strawberries in profusion, with cream (no sugar).

*Milk*—In moderation. Cream, butter, buttermilk, and all kinds of cheese freely.

*Bread.*—Only that made from wheat gluten flour. From gluten a number of palatable breads, rolls, pancakes, fritters, crullers, griddle-cakes, mushes and puddings are made, rendering ordinary bread unnecessary.

*Nuts*—Almonds, walnuts, Brazil nuts, filberts, pecan nuts and butternuts. They should be freely salted.

*Pastry.*—None, unless made from gluten flour, without sugar.

*Eggs.*—Plenty of them. If boiled, let the time not exceed two minutes. Use in any way except in sweet omelettes and custards.

Pickled codfish, with eggs. Scrambled eggs, with chipped beef.

*Coffee and Cocoa.*—Moderately, with glycerine (no sugar). Cereal coffee particularly recommended. Tea objectionable.

*Spirits and Liqors.*—None, and no wine, except claret, Burgundy, Rhine, or other acid varieties, in moderation. Claret preferred. No malt liquors.

Eat slowly, and in moderate quantities. Take as little liquid as possible during meals, and throughout the day.

The tendency is to a dry skin, and perspiration being highly important, frequent warm baths are advised; the evening is the best time. Cold or tepid baths may be taken with advantage in the morning, exercising afterward, to restore the circulation. Turkish baths are also recommended once or twice a week, if approved by your physician. Exercise as freely as possible in the open air, and sleep eight hours of the twenty-four.—*Medical and Surgical Reporter.*

